# Supplementary figures and images for: A dual-plane fluoroscope to track joint kinematics during dynamic daily activities
Source: PLoS One. 2025 Jul 24;20(7):e0328351. doi: 10.1371/journal.pone.0328351 (PMC12289030; doi:10.1371/journal.pone.0328351)

ventral

Markerset IfB

dorsal

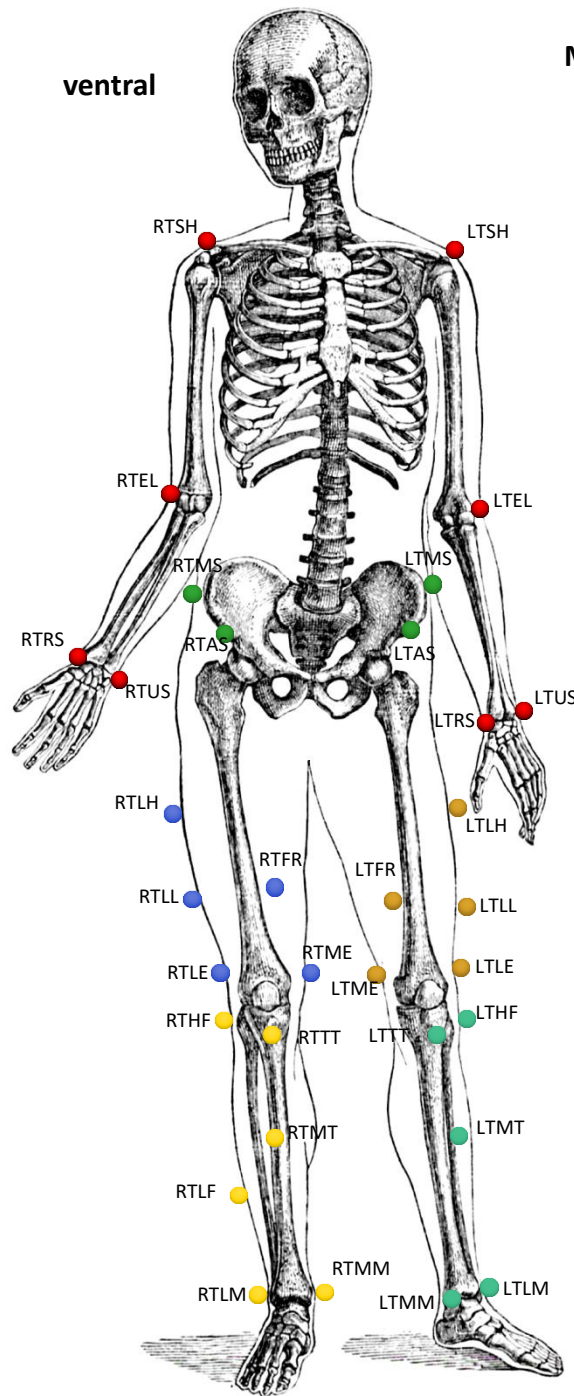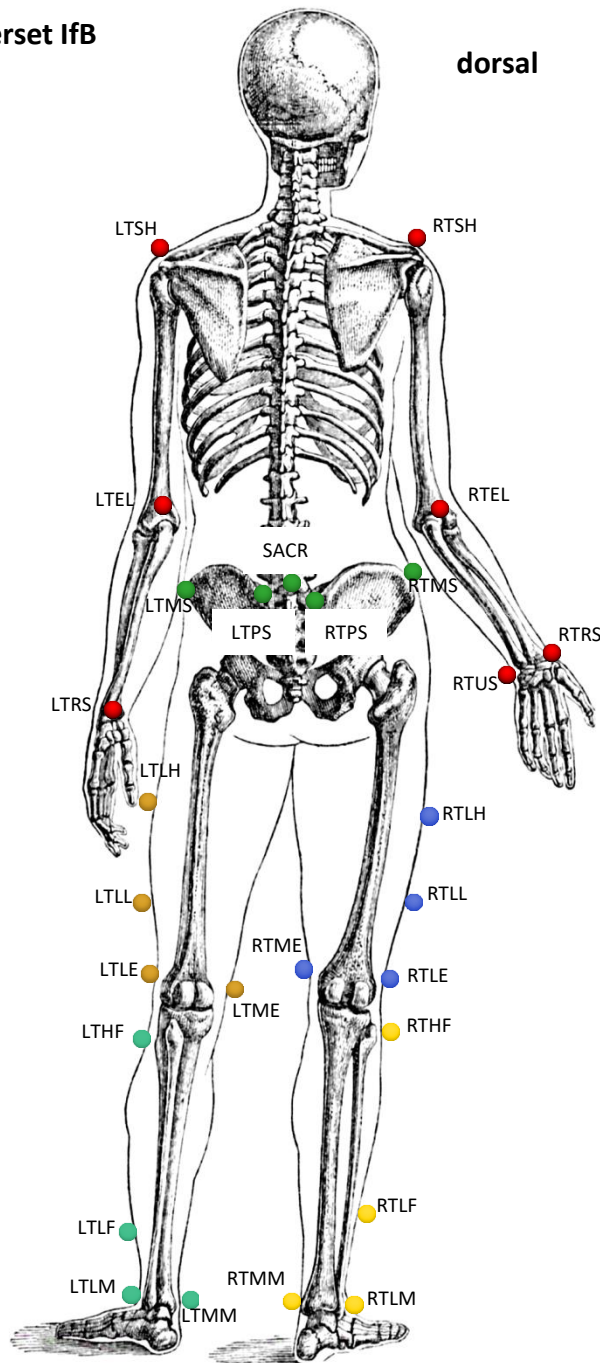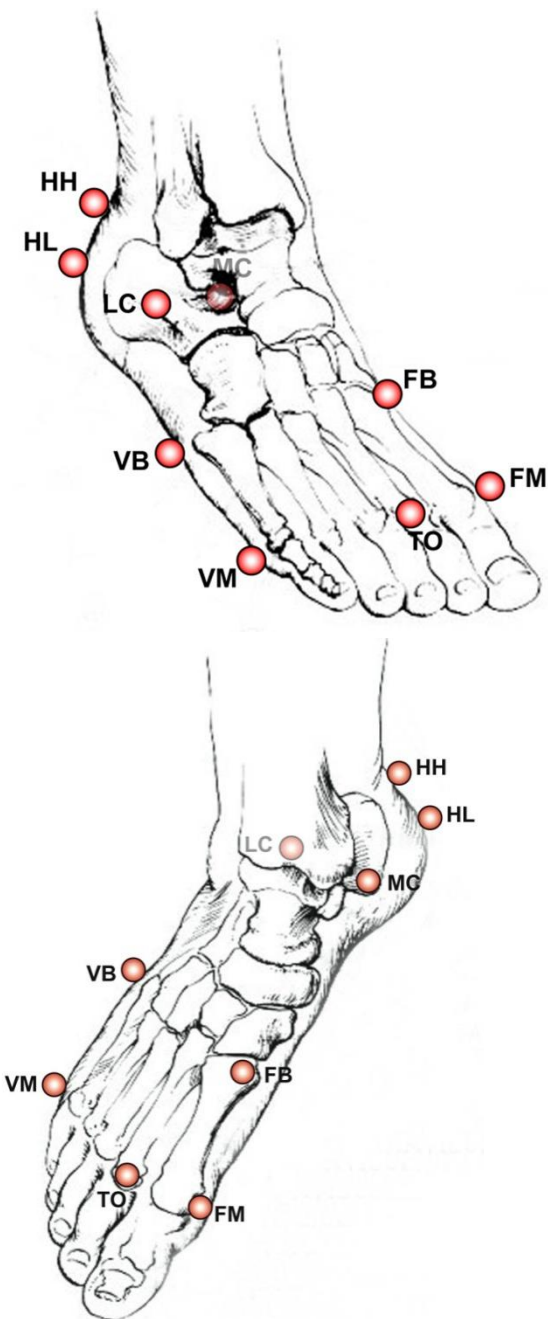

Supplement: S1 Fig — (PDF) [file pone.0328351.s001.pdf]

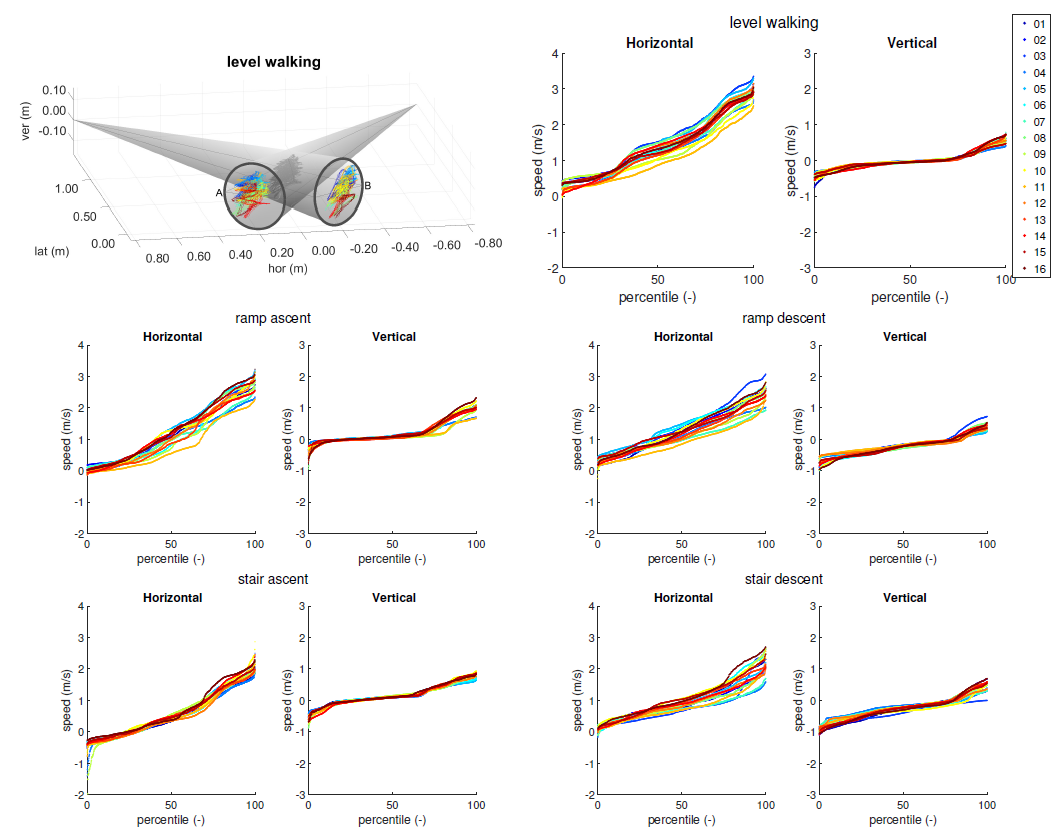

Supplement: S2 Fig — For level walking, stair ascent, and ramp ascent, the subjects were moving from left to right, while for stair descent and ramp descent, the subjects were walking from right to left. (PNG) [file pone.0328351.s002.png]

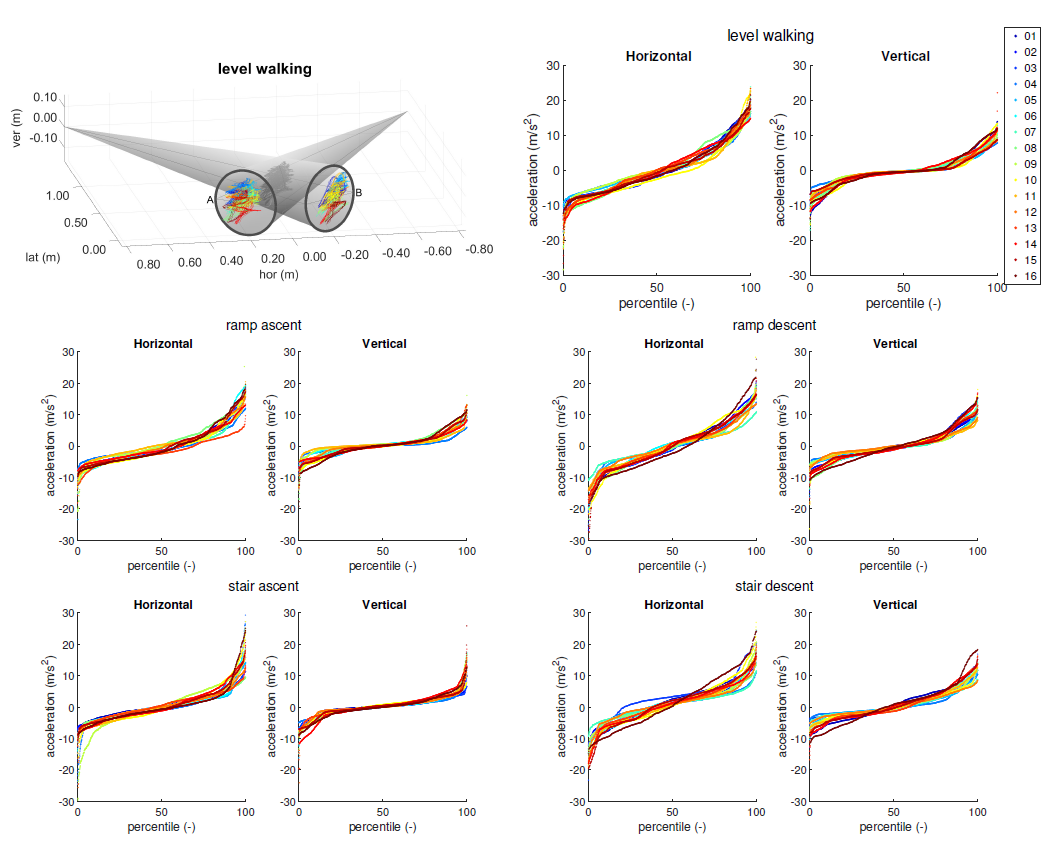

Supplement: S3 Fig — For level walking, stair ascent, and ramp ascent, the subjects were moving from left to right, while for stair descent and ramp descent, the subjects were walking from right to left. (PNG) [file pone.0328351.s003.png]

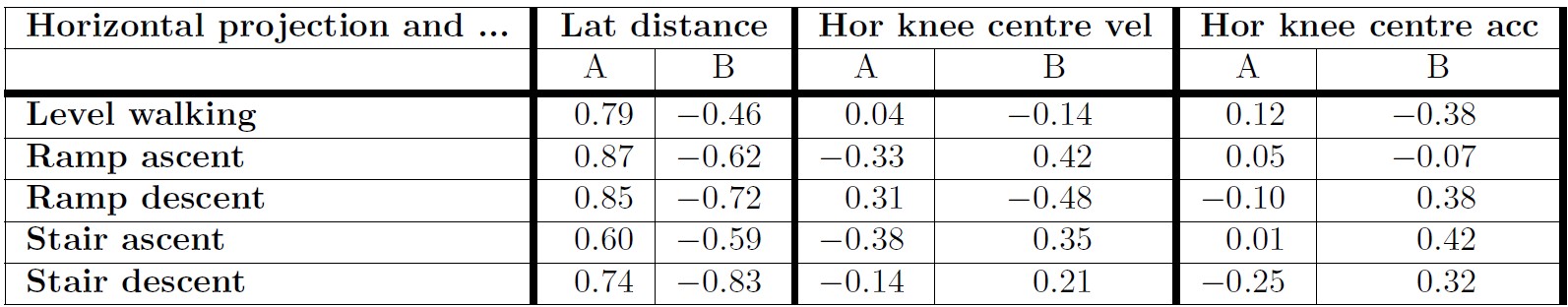

Supplement: S1 Table — (JPG) [file pone.0328351.s004.jpg]
