# Supplementary material for: A dual-plane fluoroscope to track joint kinematics during dynamic daily activities
Source: PLoS One. 2025 Jul 24;20(7):e0328351. doi: 10.1371/journal.pone.0328351 (PMC12289030; doi:10.1371/journal.pone.0328351)
Supplement: Attachment — Submitted filename: Responses to Reviewers - aplanta.pdf [file pone.0328351.s006.pdf]

Responses to Reviewers

Reviewer #1: This paper introduces the novel iteration of a double fluoroscope mounted on a robot to measure accurate knee kinematics during dynamic activities. The study goal is to present the device and validate the knee position tracking during various activities with healthy participants. Such system is very relevant for the field and is very promising in terms of future applications. This is a high quality paper, well written and easy to follow. However, I still have two major concerns, the first one regarding the metrics used to assess the quality of the tracking and the second one on the shared data.

We thank the reviewer for his valuable input and constructive feedback. Answers are below in a point-to-point manner in red. Changes made to the manuscript are highlighted in yellow, the page and line numbers mentioned refer to the adapted manuscript with tracked changes (Manuscript_adapted_TC).

Major concerns:

- Metrics - The metric used is the distance between the knee centre and the centre of the field of view (FoV) of each fluoroscope. The study shows multiple timepoints where the knee centre is on the edge of one or the other fluoroscope's FoV. If the knee centre is at the edge of the FoV, the image measured by the fluoroscope will not contain the whole view of the knee, e.g. a femoral condyle or part of the tibial plateau may be missing. And, if this is the case on both views at the time the view of the anatomical structure may be quite limited. My concern is the following: is it possible to estimate accurately the pose of the femur or tibia with only partial view of the bone on one or two images? If this is the case, the authors should elaborate on this in the manuscript with appropriate references. If this is not the case, the results may overestimate the quality of the tracking and authors may have to use a metric estimating whether the volume of the knee is within the volume of acquisition during the various tasks, instead of 2 separate 2D metrics.

The reviewer raises an important issue that we have discussed intensively within our team. Based on the input from our colleagues who are working on the image-processing, it was decided that partial view of the knee (at least half of the knee is in the field of view) should be sufficient for the intended analysis of the data. Further, a similar metric was used in a previous publication using a single-plane fluoroscope (List et al 2017), in which they reconstructed the knee kinematics using the partial view of the knee including its centre A next study including imaging is however necessary to ensure this also holds in practise for our dual-plane fluoroscope. To better inform the reader about the possible limitations of the chosen metric, we added the following sentences within the discussion:

Page 9, Lines 308-313:

Thus, it remains to confirm that the resulting image quality – possibly affected by non-complete captures of the knee and vibrations on the structures carrying the X-ray system – would be sufficient to perform kinematic reconstruction of the knee with the required accuracy. A previous publication using a single-plane fluoroscope indicated that a partial image of the knee including its centre was sufficient to reconstruct the knee kinematics [9]

- Data - The authors made a great effort in publishing the data, however, they are currently in proprietary format and requires MATLAB licences to be used. This goes against the FAIR (Findability, Accessibility, Interoperability, and Reusability) Data Principles, please update the data format to match these recommendations.

Our .mat files can also be opened and processed within Python, please see for example https://www.askpython.com/python/examples/mat-files-in-python. Due to the nested structure of the .mat files we consider this clearer for the interested reader compared to providing .csv or .tx

Minor concerns & Comments:
General questions:

1. Although this may not be the focus of research, could the authors elaborate on potential applications of this system to other joints?

A paragraph on possible elaboration of the potential application of the system to other joints have been added in the discussion.

Page 9, lines 298-306:

Additionally to tracking slow and fast knee movements in TKA patients, the use of the tDPF could be extended to other movement disorders such as cerebral palsy (CP). By tracking the knee during crouch gait, insight can be won into CP subjects' tibio-femoral kinematics which could support treatment planning. The application area of the tDPF could also be extended to other joints of the human body – e.g. patello-femoral joint, hip, or shoulder – due to the range in vertical travelling distance. Thereby enabling not only research towards implant development and healthy kinematics but also pathogenesis of for example shoulder problems in wheelchair users.

2. The study mentions the tibio-femoral joint, could this tool be used to track the patello-femoral joint as well? If so, it would be interesting to mention it for reader not familiar with dual fluoroscopy.

Within our current manuscript we focused on the application on the tibio-femoral joint. Of course the system could also be used to track the patella-femoral joint, this might however require other tracking optimizations. We added the possible application to the patella-femoral joint in the discussion.

See above.

3. What is the weight of the full system? Did you have to reinforce the screed of the room where the system is?

Approximately 642kg (source carriage) and 663kg (intensifier carriage). The system is mounted on concrete.

4. Since the emitters is on one side of the walkway, is possible to measure either knee when walking or can the system only measure the knee that is closest/farthest to the emitter?

Per walk, the closest knee to the image intensifiers can be measured. We added this information within the methods section.

Page 3, lines 59-60:

The closest knee to the image intensifiers is measured.

5. The title and legend of the figure seems to be missing. In PlosOne they are generally reported after the paragraph where they are mentioned first.

The title and legends of the figures are now added within the manuscript.

Specific remarks:

6. P2L26: "our system only used the real-time estimates of the joint to achieve its tracking". Do you mean "joint centre"? if so, please specify to help the understanding.

Here, we specifically mean the joint and not the joint centre, as we describe the single-plane fluoroscope's tracking, not the dual-plane fluoroscope's tracking nor the metric used in this manuscript.

7. P2L30: "tasks with high variability between trials", could you provide 1 or more examples to clarify what you mean?

An example has been added.

Page 2, line 30:

… , e.g. TKA patients performing level walking.

8. P2L31: The "However" seems irrelevant, please rephrase.

We think the "however" is relevant, as in the previous paragraphs the advantages of the single-plane fluoroscope are explained, while in this paragraph the limitations are elaborated. We however rephrased one of the above sentences to make that clearer.

Page 2, lines 24ff:

Our system only used the real-time estimates of the joint to achieve its tracking, in contrast to other tracking methods that required one or more reference trials to be recorded before the tracking could be used in actual measurements. This avoids the aforementioned reference trials, and tracking is possible immediately. Additionally, each trial is tracked independently from the others, which allows tracking of challenging tasks with high variability between trials, e.g. TKA patients performing level walking.

However, the automated single-plane fluoroscope could only track slow gait speeds, as it was limited by the traction between its wheels and the ground.

9. P3L99: How was the threshold of 50mm chosen?

This threshold was chosen heuristically.

10. P4EQ1: The admissible range of the motor torque mentioned L123 is not reported.

We added the information in the formula.

11. P5L165: The four hours include also preparation of setup and cleaning or just the time with the participant?

The four hours included just the time with the participant, this information has been added to the method section.

Page 5, lines 169-170:

…, preparation not included.

12. P7L242: ”Therefore there need to be another effect causing differences in performance tracking between subjects”. Does the authors have some hypotheses on the other potential sources of errors?

The other potential source of error is elaborated in the following paragraphs.

13. Figure 5 represent the distribution in percentage of the distance to the centre of the FoV?

Figure 5 shows the distribution (in percentiles) of the absolute distance between the knee centre and FoV centre per task and subject.

14. Are figure 10,11,12,13 mentioned in the manuscript?

All figures are now referenced within the manuscript. We removed the previous Figs 9 and 12. Figs 10, 11 and 13 are now called S1, S2 and S3 Fig.
