# Supplementary material for: A dual-plane fluoroscope to track joint kinematics during dynamic daily activities
Source: PLoS One. 2025 Jul 24;20(7):e0328351. doi: 10.1371/journal.pone.0328351 (PMC12289030; doi:10.1371/journal.pone.0328351)
Supplement: Attachment — Submitted filename: Responses to Reviewer 2 - aplanta.pdf [file pone.0328351.s007.pdf]

Responses to Reviewer 2

We thank the reviewer for his valuable input and constructive feedback. Answers are below in a point-to-point manner in red. Changes made to the manuscript are highlighted in yellow, the page and line numbers mentioned refer to the adapted manuscript with tracked changes (Revised Manuscript with Track Changes).

Reviewer #2: The authors present a first-of-its-kind dual fluoroscope setup for tracking the entire gait cycle during walking and stair ascent/descent. The experimental advance is noteworthy, and the authors are commended for developing an impressive setup for tracking knee joint motion in healthy participants and later in patients following total knee arthroplasty (TKA). The manuscript provides sufficient detail to describe the system setup and, if others had the resources, replicate the work. (Of particular note is the description for the tracking of knee joint position and controller.)

On the other hand, the potential advance of this setup is clouded by the lack of gold standard measurements to compare tracking results against. Knee position tracking is an understandable target, but the authors' setup may perform worse than single plane system or static dual fluoroscopy systems due to movement of the emitters and intensifiers.

Furthermore, the knee centre location was not observed on fluoroscope images, further shadowing the potential advancement described in the manuscript. For example, knee joint positions on the trailing edge of the fluoroscopes will have very little bone in the image for registration, but knee joint positions towards the leading edge will have much of the bone available for image registration.

We agree with the reviewer that direct validation against fluoroscopic images would strengthen confidence in the tracking setup. However, image acquisition was not feasible for ethical reasons during this proof-of-concept study, we explicitly clarified in the text (Page 9, Lines 311-312). This study was intended as a preliminary validation of the mechanical and tracking performance before future X-ray-based validation.

Another concern is the simulated cones for determining the fluoroscopes' overlap. While the authors are commended for performing this calculation, the edges of the image intensifiers do not always yield usable image data for image registration. An object with the simulated cones dimensions, or close to it, imaged with both fluoroscopes, even statically, would lend confidence to the authors' calculations. In the manuscript, all references to staying in the fluoroscopes' FOV needs to include that the FOV was simulated.

We have now consistently clarified throughout the manuscript (abstract, methods, results, and conclusion) that the FOVs were **simulated** and that **no X-ray images were captured** during the study.

Abstract

Page 2, Line 52

Page 5, Line 138

Page 6, Lines 208, 211, 212, 220

Page 7, Lines 238, 240, 243

Page 8, Fig 5, Table 2

Page 10, Line 334

Also of concern, but less so, the references to ‘all gait speeds’ refers to a narrow, self-selected band of speeds in healthy participants. Patients following TKA may walk at slower speeds and, more importantly, with higher variability, which, again, brings into question the advancement described by the authors. While the authors acknowledged the lowest gait speed for a single trial, separating the trials into different groups would have made their point about variable speeds much stronger.

We acknowledge the reviewer’s concern and agree that our participant cohort represents a limited speed spectrum compared to clinical populations. However, we would like to clarify that our reference to “all gait speeds” is **clearly contextualized within the manuscript** as self-selected speeds by healthy participants. Our intent with this phrase is to demonstrate that the system places **no constraints** on participant speed during level walking, ramp climbing, and stair climbing, unlike previous moving fluoroscopes that were mechanically limited to slow gait.

While we did not group trials by speed for sub-analysis, the observed range — from below 1 m/s to well above average walking speed — **encompasses the typical spectrum seen in both healthy and post-TKA individuals**, as cited in our discussion. The key advancement, in our view, lies not in the specific speeds tested, but in the **technical capability of the system** to track across this full range without pre-programmed trajectories or speed limitations.

We believe the current phrasing and data presentation sufficiently support this point, and no changes to the manuscript were made in this regard.

Please change all references to ‘tracking’ with ‘knee joint position tracking’, because in the field of fluoroscopy tracking often refers to model-based tracking. And accuracy of tracking typically refers to comparisons to a gold standard for joint translations and rotations.

We appreciate the reviewer’s concern and fully agree that in the context of fluoroscopy, "tracking" can sometimes refer specifically to model-based tracking. However, in this manuscript, the term “tracking” is consistently and clearly used as referring to the **real-time movement of the imaging system based on optical position estimates of the knee joint**. This is a central and unambiguous concept throughout the paper.

To prevent potential ambiguity, we have now clarified this distinction in the Introduction.

Page 2, Line 25

“…joint position…”

We believe this clarification makes the intended meaning explicit, while avoiding the stylistic burden and redundancy of replacing all instances of “tracking” with “knee joint position tracking.” Therefore, the original terminology has otherwise been retained.

Specific Comments

Abstract

As referenced in the general comments, please clarify that the FOV was simulated, and that no images were acquired.

Abstract

“(no X-ray images were captured in this study)”

Introduction

No comments. Well written.

Methods

For lines 82-85, “As the radius of the intensifier sensor is 158mm, this setup (Fig 2) results in a capture volume with a length of 305mm in horizontal direction and a length of 445mm in lateral direction, and a length of 248mm in vertical direction at the intersection of the beam cone symmetry axes …”, were these theoretical values? Or experimental measures? Oftentimes the image intensifiers do not have usable image data all the way to the edge. Either way, the authors are commended for performing and reporting these calculations/measurements.

Page 3, Line 83-84

“(experimentally measured)”

On line 87, “The knee centre is tracked using an active marker tracking system (Fig 3):….” Was the active marker placed at a standard distance up the thigh? And did the tracking include an directionality to determine if the knee was bent or straight?

Page 3, Lines 91-92

”… at a comfortable and secure location on the thigh. Directionality of knee flexion/extension was not included in the tracking.”

The ‘Optical knee tracking’ section is noteworthy for the details provided on the experimental implementation. The text describes potential ‘rejected’ positions. Do the authors have an estimate for how often this occurred?

Typically below 10 percent.

Three to four hours for acquisition seems rather long if no fluoroscope images were acquired. As alluded to the in the last comment, did the authors experience setbacks with the system that would not be expected in a traditional motion capture setup?

The total duration included participant preparation, marker placement, and calibration. Some minor setup adjustments were needed that would not be required in conventional motion capture setups. These adjustments typically took up to 30 minutes.

Results & Discussion

Could the authors describe the results in Figure 5 in more detail? The text merely states that the tracking varied substantially between subjects. Also of note in Figure 5, it’s not clear what the y axis is plotting from the caption. Distance from the projected knee joint position to the center of the image intensifier?

Page 8, Fig 5

“…to the FOV centres…”

We respectfully note that the main finding illustrated by Figure 5 — **substantial inter-subject variation in tracking performance within each task** — is already clearly described in the manuscript.

The purpose of this figure is to highlight that, despite similar task dynamics across participants, the projected knee centre positions vary between individuals, supporting our conclusion that geometric factors (e.g., lateral offset) influence tracking accuracy more than kinematic variability. We believe the level of detail currently provided appropriately reflects the intended message of this figure.

Lines 278-279, "leading to the negative values in the vertical plots of Fig 5." The values in the plots look all positive. Are the authors referring to the positions plotted on the image intensifiers? Also, why did this not lead to the knee joint center position being outside the FOV, if the knee centre was lower the safety distance?

Page 8, Line 282

This was a typo. Corrected to refer to **Fig 8**, not Fig 5.

The vertical axis was constrained by a safety limit to avoid collision with floor fixtures. The knee could remain within the simulated FOV even at this lower bound.

Could the authors explain this statement in greater detail? Lines 307-308, "This study is limited by the fact that the X-ray system was not used due to ethical considerations." Isn't the point of the system to use x-rays? It would be understandable to assess tracking of the knee joint position to confirm performance prior to exposing participants to x-rays. But it's not clear if that was what the authors are implying.

Page 9, Lines 311-312

"… as the purpose of this study was to confirm tracking performance prior to exposing participants to X-rays."

Figures and Tables

Figure 1 is a nice zoomed-out view of the system, but, being the first figure, labels for the different components would benefit the readers who are not as familiar with fluoroscope systems.

Labels added.

The authors are applauded for the impressive polar plots in Figure 6 that clearly demonstrated the results.

For Figures 5, 6, 8, 10, and 11, it would help the reader interpret the images to know which direction the participants were moving in. This was mentioned in the text but not in the captions nor figures.

Added to captions of Fig 5, 6, 8, 10, and 11:

"For level walking, stair ascent, and ramp ascent, the subjects were moving from left to right, while for stair descent and ramp descent, the subjects were walking from right to left."

Conclusions

Same as the abstract, please insert 'simulated' prior to FOV, and clarify that no x-ray images were acquired. Mentioning that the knee joint position was tracked with skin markers is also necessary.

Page 10, Line 335

"… using skin marker measurements of the knee joint position."

The last statement needs to be removed or clarified, as the high variability is between tasks and not necessarily between trials. Between trials implies the same participant completed the task differently, which the authors did not necessarily show.

We appreciate the reviewer's observation and agree that the current dataset was not designed to explicitly quantify trial-to-trial variability. However, we respectfully maintain the phrasing, as the key point being made refers not to observed variability in this cohort, but to the **system's design principle**: our tracking method operates **independently for each trial** and does not require prior recordings or reference trajectories. This technical approach is specifically intended to handle **potential variability between trials**, which is known to be a relevant factor especially in clinical populations (e.g., TKA or neurological conditions), even if not directly measured in this study.

To clarify this distinction, we have revised the conclusion to better reflect that our statement refers to the **capability** and **robustness** of the system, rather than a finding demonstrated in the presented results:

Page 10, Lines 340-344

"By operating independently on each trial — without relying on prior trial data — our system is inherently suited to accommodate inter-trial variability, which is especially relevant in clinical contexts where such variability is common. While not directly assessed in this study, this robustness is a core feature of the tracking capability and supports broader applicability in patient populations."
